# Supplementary material for: Interferon-β Overexpression in Adipose Tissue-Derived Stem Cells Induces HepG2 and Macrophage Cell Death in Liver Tumor Organoids via Induction of TNF-Related Apoptosis-Inducing Ligand Expression
Source: Int J Mol Sci. 2024 Jan 22;25(2):1325. doi: 10.3390/ijms25021325 (PMC10816756; doi:10.3390/ijms25021325)
Supplement: Supplementary file 1 [file ijms-25-01325-s001.zip › ijms-2819897-supplementary.pdf]

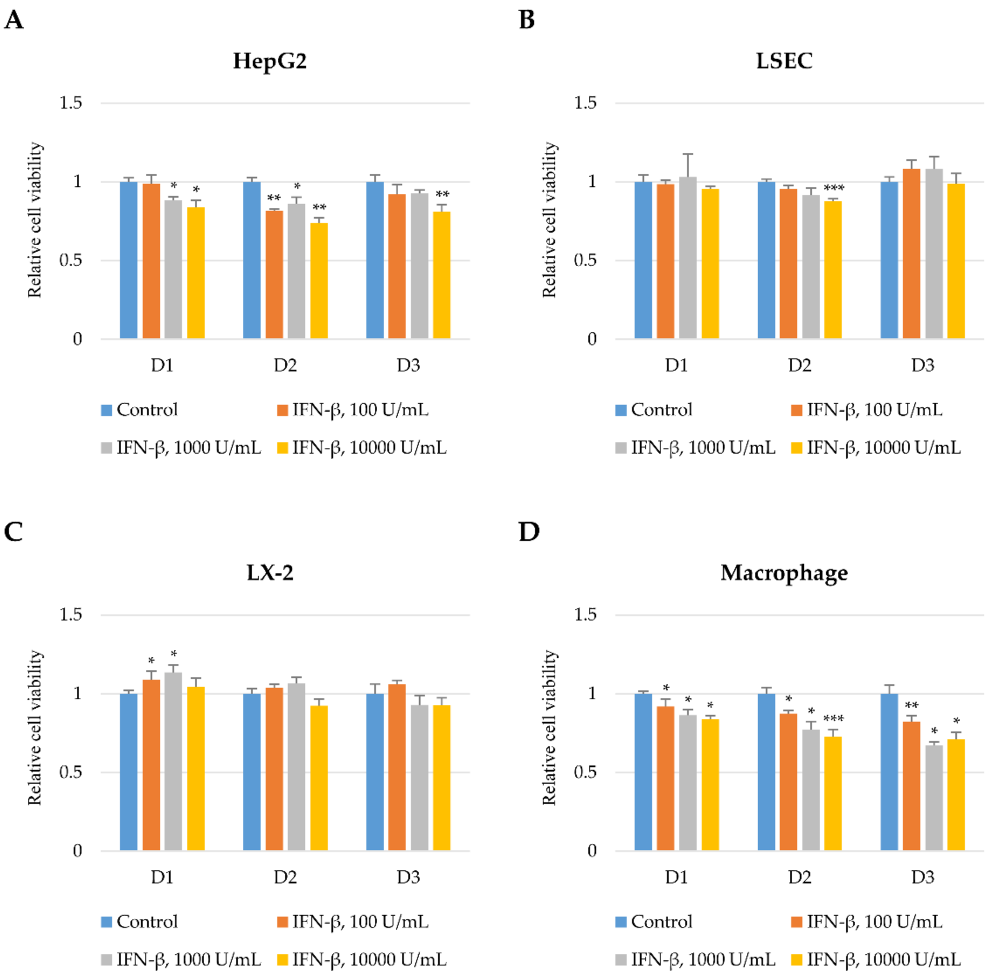

Figure S1. Relative cell viabilities in IFN-β-treated HepG2 (A), LSEC (B), LX-2 (C), or macrophages (D). HepG2, LSECs, LX-2, or macrophages were treated with 100-10000 U/mL of IFN-β, and cell viability was analyzed for 3 days. *$p < 0.05$, **$p < 0.01$, and ***$p < 0.001$.

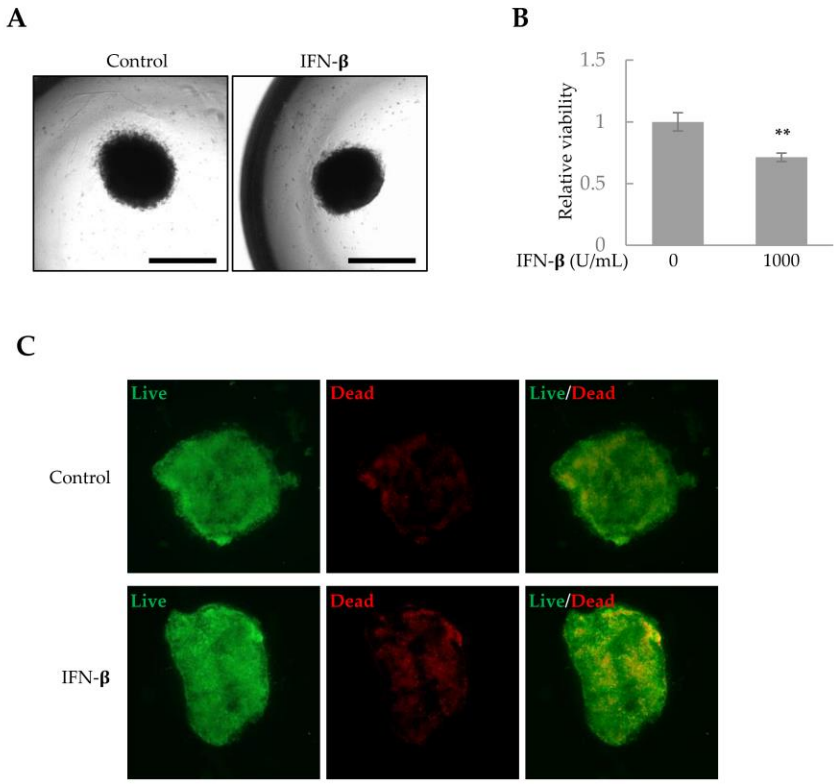

Figure S2. Effect of IFN-β on Huh-7-based liver tumor organoids. On day 3, Huh-7-based liver tumor organoids were treated with IFN-β for an additional day. (A) Representative images after IFN-β treatment. Huh-7-based liver tumor organoids exposed to IFN-β retained their spherical shape. Scale bar: 1 mm. (B) Decreased viability of Huh-7-based liver tumor organoids treated with IFN-β. **$p < 0.01$. (C) Cell death induced by IFN-β in Huh-7-based liver tumor organoids. IFN-β-induced cell death was evaluated using a live (calcein-AM) and dead (ethidium homodimer-1) assay.

Table S1. Primer sequences used in this study.

Gene name	Sequence (5'-3')		Accession #
GAPDH	Forward	CAAGGCTGAGAACGGGAAGC	NM_001256799.3
	Reverse	AGGGGGCAGAGATGATGACC	
AFP	Forward	AGTGAGGACAAACTATTGGCCT	NM_001354717.2
	Reverse	ACACCAGGGTTTACTGGAGTC	
ALB	Forward	TTTATGCCCCGGAACTCCTTT	NM_000477.7
	Reverse	AGTCTCTGTTTGGCAGACGAA	
CYP3A4	Forward	AAGTCGCCTCGAAGATACACA	NM_001202855.3
	Reverse	AAGGAGAGAACACTGCTCGTG	
CYP2D6	Forward	TGGCAAGGTCCTACGCTTC	NM_000106.6
	Reverse	GCCACCACTATGCACAGGTT	
G6PC1	Forward	CTACTACAGCAACACTTCCGTG	NM_000151.4
	Reverse	GGTCGGCTTTATCTTTCCCTGA	
HNF4a	Forward	CACGGGCAAACACTACGGT	NM_178849.3
	Reverse	TTGACCTTCGAGTGCTGATCC	
UGT2B7	Forward	GATCCCAACAACTCATCCGCT	NM_001074.4
	Reverse	CAGCAGCTCACTACAGGGAA	
AXIN2	Forward	AGCTTACATGAGTAATGGGG	NM_004655.4
	Reverse	AATTCCATCTACACTGCTGTC	
iNOS	Forward	GTGCAAACCTTCAAGGCAGC	NM_000625.4
	Reverse	CTTGGCCATCCTCACAGGAG	
IL-1β	Forward	CAGGCTGCTCTGGGATTCTC	NM_000576.3
	Reverse	GTCCTGGAAGGAGCACTTCAT	
TNF-α	Forward	CCCATGTTGTAGCAAACCCT	NM_000594.4
	Reverse	TGAGGTACAGGCCCTCTGAT	
IL-10	Forward	TTCGAGATCTCCGAGATGCC	NM_000572.3
	Reverse	AGTTCACATGCGCCTTGATG	
TGF-β	Forward	AAGTGGACATCAACGGGTTC	NM_000660.7
	Reverse	GTCCAGGCTCCAAATGTAGG	
CD206	Forward	GTGATGGGACCCCTGTAACG	NM_002438.4
	Reverse	CTGCCCAGTACCCATCCTTG	
CD31	Forward	AACGGAAGGCTCCCTTGATG	NM_000442.5

	Reverse	TAAGAACCGGCAGCTTAGCC	
LYVE1	Forward	GAGCAAAAAGGCGAACCAGC	NM_006691
	Reverse	CAACCCAGCCATAGCTGCAAG	
VEGF-A	Forward	ACATCACCATGCAGATTATGCG	NM_003376.6
	Reverse	ACCGGGATTTCTTGCGCTTT	
VEGFR2	Forward	ACCGGCTGAAGCTAGGTAAG	NM_002253.4
	Reverse	CGATGCTCACTGTGTGTTGC	
α-SMA	Forward	GACAATGGCTCTGGGCTCTGTAA	NM_001613.4
	Reverse	CTGTGCTTCGTCACCCACGTA	
COL1A1	Forward	CAGGAGGCACGCGGAGTGTG	NM_000088.3
	Reverse	GGCAGGGCTCGGGTTTCCAC	
COL3A1	Forward	TCCCGGTCCTGCTGGTTCCC	NM_000090.4
	Reverse	ATGGCAGCGGCTCCAACACC	
COL5A3	Forward	AGTTTCCCGCGGACCC	NM_015719.4
	Reverse	ACATCCACAGGATCGGCCT	
LAMA1	Forward	CTTCCTGAAAGGCGGCTACA	NM_005559.4
	Reverse	TGTGCTTCCTCACGATCACC	
